# Cognitive neuroscience of human counterfactual reasoning

**DOI:** 10.3389/fnhum.2015.00420

**Published:** 2015-07-23

**Authors:** Nicole Van Hoeck, Patrick D. Watson, Aron K. Barbey

**Affiliations:** ^1^Psychology and Educational Sciences, Vrije Universiteit BrusselBrussels, Belgium; ^2^Decision Neuroscience Laboratory, Beckman Institute for Advanced Science and Technology, University of IllinoisUrbana, IL, USA; ^3^Department of Internal Medicine, University of IllinoisChampaign, IL, USA; ^4^Department of Psychology, University of IllinoisChampaign, IL, USA; ^5^Department of Speech and Hearing Science, University of IllinoisChampaign, IL, USA; ^6^Neuroscience Program, University of IllinoisChampaign, IL, USA; ^7^Carle R. Woese Institute for Genomic Biology, University of IllinoisChampaign, IL, USA

**Keywords:** counterfactual thought, mental simulation, adaptive behavior, clinical disorders

## Abstract

Counterfactual reasoning is a hallmark of human thought, enabling the capacity to shift from perceiving the immediate environment to an alternative, imagined perspective. Mental representations of counterfactual possibilities (e.g., imagined past events or future outcomes not yet at hand) provide the basis for learning from past experience, enable planning and prediction, support creativity and insight, and give rise to emotions and social attributions (e.g., regret and blame). Yet remarkably little is known about the psychological and neural foundations of counterfactual reasoning. In this review, we survey recent findings from psychology and neuroscience indicating that counterfactual thought depends on an integrative network of systems for affective processing, mental simulation, and cognitive control. We review evidence to elucidate how these mechanisms are systematically altered through psychiatric illness and neurological disease. We propose that counterfactual thinking depends on the coordination of multiple information processing systems that together enable adaptive behavior and goal-directed decision making and make recommendations for the study of counterfactual inference in health, aging, and disease.

## Introduction

“Without considering alternatives to reality, we must accept the past as having been inevitable and must believe that the future will be no different from the past. The generation of counterfactuals gives us the flexibility in thinking about possible futures and prepares us better for those futures”

(Johnson and Sherman, 1999, p. 150).

Humans have the remarkable ability to infer how an event might have unfolded differently, without directly experiencing this alternative reality. We employ such counterfactual reasoning to make sense of the past, plan courses of action, make emotional and social judgments, and guide adaptive behavior. In this review article, we introduce an integrative cognitive neuroscience framework for understanding the psychological and neural foundations of counterfactual thought, drawing upon cognitive and neuroscience evidence to suggest that counterfactual thinking depends on an integrative network of systems for affective processing, mental simulation, and cognitive control.

We begin by surveying the psychological literature on counterfactual reasoning, followed by a review of the neuroscience literature on affective processing, mental simulation, and cognitive control networks supporting counterfactual thought. Finally, we review evidence to elucidate how these mechanisms are systematically altered in psychiatric illness and neurological disease and draw conclusions about the importance of counterfactual thought for understanding adaptive behavior and goal-directed decision making.

## Stages of Counterfactual Thought

**Figure 1:**
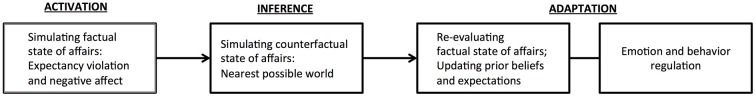
**Stages of counterfactual reasoning: a schematic overview**.

We propose that counterfactual reasoning depends on three processing stages: *Activation*, *Inference*, and *Adaptation* (see Figure 1; e.g., Byrne, 2002; Epstude and Roese, 2008; Barbey et al., 2009). To illustrate these stages, consider the example of receiving a rejection letter for a new job. The letter would *activate* prior memories (e.g., the process of submitting an application and interviewing for the position) and elicit a mental simulation of the event (e.g., representing relevant knowledge and expectations; for example, the job requirements). As this activation spreads, it triggers mental simulations of similar situations (previous applications), allowing one to *infer* how this scenario would have played out under different circumstances (e.g., if one had spent more time preparing for the interview; i.e., the counterfactual event). These nearby counterfactual simulations influence ones interpretation of the factual experience (e.g., the committees decision was caused by my failure to adequately prepare for the interview) and promote *adaptive behavior* that guides future planning and problem solving. The process of counterfactual reasoning therefore supports inferences about the factual event that guide understanding, modulate emotional responses, and provide the basis for future planning and decision-making.

Our proposal is that the coordination of systems for: (i) mental simulation; (ii) inference; and (iii) learning and adaptation together enable counterfactual reasoning. In the following sections, we review each of these processes and survey evidence from psychology and neuroscience to elucidate their contributions to counterfactual reasoning.

### Activation of Mental Simulations

Counterfactual thoughts are automatically employed in response to real world experiences, in particular those situations where negative emotions are linked to violations of expectations and motivations (e.g., an unsuccessful job-application), in the form of implicit or explicit goal failures (e.g., answering the interview questions weakly), or close calls (physical, temporal or numerical proximity; e.g., being the runner-up) trigger counterfactual thoughts, representing a probable alternative state of affairs (Kahneman and Tversky, 1982; Davis et al., 1995; Sanna and Turley, 1996; Roese and Olson, 1997; Mcgraw et al., 2005; Epstude and Roese, 2008; Kühberger et al., 2011).

We propose that counterfactual thought depends on mental models of alternative possibilities represented in the form of mental simulations (Damasio, 1989; Barsalou et al., 2003a,b; Markman et al., 2009). Simulations provide the basis for constructing mental models of events and of imaging alternative realities “if only” different decisions were made or actions taken. For example, the counterfactual inference that “If I was to vacation in Europe rather than continue writing, then I wouldn’t be this stressed” activates a mental simulation, which represents relevant agents (e.g., the author), objects (e.g., museums), actions (e.g., sightseeing), mental states (e.g., freedom) and background settings (e.g., Florence). These simulations provide the basis for evaluating the consequences of the real and alternative courses of action, with the simulation of the author “vacationing in Europe” resulting in being more relaxed but not completing the article.

Our proposal shares much in common with previous frameworks of counterfactual reasoning, emphasizing the generative/constructive nature of counterfactual thought. For example, norm theory suggests that such reasoning is driven by simulations of previously encoded exemplars (Kahneman and Tversky, 1982; Kahneman and Miller, 1986). This theory emphasized the role of counterfactual thought in *reframing* such scenarios—generating alternative possibilities that change the norms (and expectations) used to interpret a state of affairs. The Model Theory of counterfactual thought (Byrne, 2002, 2007) emphasizes individuals’ ability to entertain multiple parallel models corresponding to alternative possibilities, and suggests that counterfactual thought is engaged to search the space of possible alternatives (e.g., in simulating permissible or forbidden actions). Building upon these frameworks, the structured event complex theory proposes that counterfactual thought engages a network of regions within prefrontal cortex (PFC) that represent alternative goals, behavioral intentions, mindsets, motivations, and self-inferences that enable behavioral change and adaptation (Barbey et al., 2009).

### Counterfactual Inference

In contrast to other types of conditional and hypothetical reasoning, an important feature of counterfactual inference is that it adheres to a “nearest possible world” constraint (Lewis, 1979, 1986; Hendrickson, 2010; Rafetseder et al., 2010, 2013; Van Hoeck et al., 2012). A counterfactual must closely model one’s own experience of the real state of the world. It ties to specific situational features and prior knowledge of the situation (i.e., it requires the fewest independent changes to the actual circumstances and be maximal coherent with prior history). This constraint differentiates counterfactual thought from less constrained fantasy or imagination (Rao and Foo, 1989; Lebow, 2000; Revlin et al., 2001; Roese and Summerville, 2005; Byrne, 2007; Hendrickson, 2010; Dehghani et al., 2012). The alternative outcome suggested by the counterfactual reflects a small deviation from reality and is therefore probable (Over et al., 2007; Epstude and Roese, 2008; Petrocelli et al., 2011).

For example, counterfactual thought often focuses on the factor that: (1) played the strongest role in the (un-)desired outcome; (2) deviated most from expectancies; or (3) is most under the participants control (i.e., “mutable” factor; McCloy and Byrne, 2000; Mcgraw et al., 2005; Byrne, 2007; Over et al., 2007; Dehghani et al., 2012; Rips and Edwards, 2013). Counterfactual thought rarely suggests alterations from natural or social laws (Byrne, 1997, 2007; Roese, 1997; Nasco and Marsh, 1999; McCloy and Byrne, 2000; Walsh and Byrne, 2004; Tykocinski and Steinberg, 2005; Dehghani et al., 2012). Furthermore, counterfactual thought is tied to the meaning and relevance of specific events rather than to general situations or tendencies. Consider an example in which one failed an exam by studying the wrong material. In this situation, the generalization that studying promotes academic achievement is unhelpful.

In addition to being tied to a specific event, counterfactual thought is often motivated by a specific individual perspective. An individuals’ implicit* belief of attainability* or self-efficacy will play an important role (Sanna, 1997; Wong et al., 2012; Dyczewski and Markman, 2012; Tyser et al., 2012; Zhang and Covey, 2014). For example, an individual who studied the wrong material for an exam would not believe they would otherwise pass, failing to endorse the counterfactual, “If I had studied the correct material, I would have passed the exam”, when they held the implicit belief that they are not smart enough to master the correct material. To this individual, the semifactual, “Even if I had studied the correct material, I would have failed the exam”, would be more realistic. Implicit belief can therefore explain why individuals sometimes alter other aspects than their own behavior (e.g., task-characteristics) when they are explicitly cued to generate counterfactuals (Pighin et al., 2011; Ferrante et al., 2013).

### Learning and Adaptation

Once the counterfactual state of affairs has been inferred, this information will be incorporated in the representation of the factual state of affairs (re-evaluation) and update prior beliefs and action-values, resulting in behavioral and affective consequences. Counterfactual inference supports adaptive behavior and is known to:

■ Enhance memory distortions, such as hindsight bias and source confusion (retrospective overestimation of the outcome’s likelihood), contributing to suboptimal decision making (e.g., investment practices; Roese and Olson, 1996; Roese et al., 2004; Kruger et al., 2005; Nestler and von Collani, 2008; Petrocelli and Sherman, 2010; Petrocelli and Harris, 2011; Strahilevitz et al., 2011; Gerlach et al., 2013).
■ Promote a relational/analytical or an expansive/creative processing style (Markman et al., 2007).
■ Enable learning from past experience (Byrne, 1997; Epstude and Roese, 2008; Smallman and McCulloch, 2012) and the formation of behavioral intentions (c.f., Functional Theory of Counterfactual Thinking; Epstude and Roese, 2008).
■ Support future planning and prediction (Roese, 1999; Barbey and Sloman, 2007; Markman et al., 2008; Smallman and Roese, 2009; Tobia et al., 2014).
■ Provide the basis for creativity and insight (Sternberg and Gastel, 1989; Gomez Beldarrain et al., 2005; Markman et al., 2007; Kray et al., 2009; Roese and Morrison, 2009).
■ Impart meaning to important life events by increasing perceptions of fate, benefit and growth (Davis et al., 1998; Koo et al., 2008; Kray et al., 2010; Teigen and Jensen, 2011).
■ Generate emotions and social ascriptions (e.g., guilt, regret, blame and relief) that are central for managing and regulating social behavior (Davis et al., 1995; Roese and Olson, 1997, 2007; Pieters and Zeelenberg, 2005; Alicke et al., 2008; Coricelli and Rustichini, 2010; Brassen et al., 2012; Miller et al., 2013).

Several interacting factors influence the behavioral and affective consequences of counterfactual inference. In the first place the counterfactual outcome *value* will be important. Is the alternative outcome better or worse (valence; upward vs. downward counterfactual) and by how much (magnitude)? This valuation will impact how an individual perceives the factual, experienced outcome and its relative value. Secondly, does the individual perceive a *favorable opportunity* to improve performance? Will a similar situation take place in the future (repeatability, e.g., midterm vs. final exams) and does the individual believe it is feasible to change the outcome: do they perceive to be in control of the situation and that the counterfactual state is attainable? What is the probability that the alternative action(s) can bring about the desired outcome? The third factor is the perspective or *processing mode* in which counterfactual states are represented. Markman and McMullen (2003) suggest in their Reflective and Evaluative Model that a counterfactual representation can be further processed in two ways: the counterfactual representation can function as a standard to which to compare the real world (evaluative mode) or the counterfactual representation can be further processed in a more concrete-experiential way without using it as a standard of comparison (reflective mode). These different modes are very similar to the distinction between imagining an event from the first- vs. third-person perspective. From a first-person perspective (similar to reflective mode), the individual imagines an event as if it is unfolding before them (the individual is part of the experience). However, when the event is imagined from a third-person perspective (similar to evaluative mode), the individual visualizes the event from an observer’s standpoint and examines the broader context (Libby and Eibach, 2011a,b; Valenti et al., 2011). This suggests that an evaluative focus takes current beliefs, traits, and goals more into account, whereas a reflective mode focuses more on sensory details of the counterfactual representation. We now turn to a discussion of how the combination among the reviewed factors (i.e., value, opportunity, and processing mode) influences counterfactual inference.

Evaluations of how a counterfactual possibility could have been better (i.e., upward counterfactual thoughts), and how we might have personally effected such an outcome (perceived attainability) often elicit an emotional and social reaction. For example, regret will increase as we feel more responsible for the outcome and as the discrepancy between the experienced and counterfactual outcome value increases (Zeelenberg et al., 1998; van Dijk and Zeelenberg, 2002; Nicolle et al., 2011b). Like feelings of regret, the social ascription of blame arises from counterfactual reasoning about an action that caused or contributed to the negative experience and that is perceived to be under the person’s control (see example about avoided car crash). However, blame demands an additional negative evaluation of the action itself (e.g., intentionally causing harm, breaking social norms, negligence; i.e., culpable control; c.f. Alicke et al., 2008).

Counterfactual thinking combined with an opportunity to improve future performance (repeatability of a similar event and belief in feasibility of different outcome), elicits *behavioral motivations* to pursue the counterfactual outcome, and modulates ones perception of control and preparedness, boosting persistence and performance (Roese and Olson, 1997; Sanna, 1997; Nasco and Marsh, 1999; Markman and Miller, 2006; Quelhas et al., 2008; Smallman and Roese, 2009; Dyczewski and Markman, 2012; Smallman, 2013; Zhang and Covey, 2014). It is assumed that the affect coupled with the experience will be *reappraised* in light of ones new feelings, and *inhibit* further counterfactual thoughts (Roese and Olson, 1997; Sanna, 1997; McMullen and Markman, 2002; Quelhas et al., 2008; Libby and Eibach, 2011b; Libby et al., 2011; Valenti et al., 2011). The reappraisal of the situation might also explain why the emotional intensity experienced during episodic counterfactual thinking is lower than during episodic past and future thinking (De Brigard and Giovanello, 2012).

However, in situations without opportunity for improvement (e.g., if the change is infeasible or the scenario cannot reoccur), upward counterfactual thinking will not be beneficial (Sanna, 1997; McMullen and Markman, 2002; Branscombe et al., 2003; Alicke et al., 2008; McCrea, 2008; Libby and Eibach, 2011a; Nicolle et al., 2011b; Dyczewski and Markman, 2012; Tyser et al., 2012; Wong et al., 2012; Zhang and Covey, 2014). Consider the case of breast cancer patients 3–4 months after diagnosis. In this group a high level of upward counterfactual thought is related to a high level of psychological distress (Gilbar and Hevroni, 2007). During this phase of the illness the shock of this life-threatening diagnosis is still being processed, for which upward counterfactuals would not be not helpful. Similar results have been found with assault-victims (Branscombe et al., 2003; El Leithy et al., 2006). When one perceives no favorable opportunities to obtain a desired outcome or implement corrective behavior, goal disengagement is an adaptive coping mechanism (Wrosch et al., 2013). As such, downward counterfactual thoughts (i.e., thoughts about how the event could have turned out worse) instead might help regulate emotions in these circumstances and promote the inference that an individual is a “lucky survivor” instead of an “unlucky victim” (Markman et al., 1993; Roese, 1994; Davis et al., 1998; Teigen and Jensen, 2011).

Markman and McMullen (2003) suggest that counterfactual information can also be processed in a more concrete, experiential manner (see also Libby and Eibach, 2011a,b). Effects of this processing match *affective assimilation* effects: the experienced affect is pulled into the direction of the counterfactual outcome. Processing an upward counterfactual in this manner will yield more positive affect (less intense negative emotions), but less behavioral benefits than evaluative upward counterfactuals. Vividly imagining a worse counterfactual outcome can instead lead to increase in negative affect, which can enhance persistence and performance to avoid this potentially worse outcome in the future (McMullen and Markman, 2000; Markman et al., 2008).

While most of the cases outlined above result in persistent behavioral change or re-evaluation of beliefs, not every instance of counterfactual reasoning will result in a persistent or positive behavioral change. Yet even in these cases, we suggest that counterfactual reasoning may influence the outcome. For example, if our counterfactual reasoning effort indicates a change in behavior would not result in a more favorable outcome, we still adapt our future behavior to this counterfactual information. Thus, we argue that counterfactual reasoning makes an obligatorily contribution to learning, by providing alternative realities from which to draw conclusions. Individuals therefore adapt to counterfactuals as well as real feedback-and whether this is ultimately helpful depends upon future experiences.

These diverse effects derive from essential characteristics of counterfactual thought, resulting from the construction of a coherent and plausible representation of a nearby alternative that is maintained alongside an accurate representation of the actual event. The engagement of attentional and executive control processes enables comparison between these parallel representations. This comparison ultimately drives the evaluation of the final outcome and its contribution to learning.

Thus, counterfactual thinking is a constructive process. It draws for content upon general semantic knowledge, and specific memories of past experiences. It requires spatial and temporal integration of the elements of experience. It utilizes working memory in the storage and manipulation of items. It requires emotional processing, and integrates motivations and goals. These higher cognitive functions interact to construct an internal representation of the target scenario.

## Neural Networks

**Figure 2:**
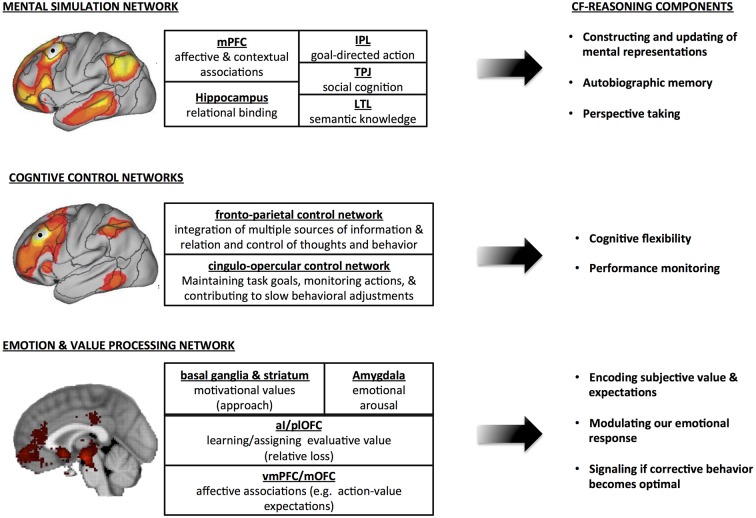
**Neural networks supporting counterfactual reasoning: a schematic overview.** CF, counterfactual; mPFC, medial prefrontal cortex; IPL, inferior partietal lobe; TPJ, temporo-parietal junction; LTL, lateral temporal lobe; aI, anterior insula; plOFC, posterior lateral orbitofrontal cortex; vmPFC, ventral mPFC; dmPFC, dorsal mPFC. For generating the brain image example of the emotion and value processing network we made use of the neursynth.org database (see also Yarkoni et al., 2011).

We review neuroscience evidence to elucidate three neural networks that together support counterfactual thought: (1) the mental simulation network; (2) the cognitive control network; and (3) the reward network (see Figure 2). As such counterfactual reasoning is supported by “networks that underlie domain general functions that cut across different psychological domains” (Barrett and Satpute, 2013). It is the *interaction* between these networks that make the complex psychological phenomena of counterfactual reasoning, including its effect on our behavior and emotions, possible. This view indicates that there is no distinctive “counterfactual reasoning” network nor that there is a one-on-one association between one of the processing stages of counterfactual reasoning we discussed above and one specific neural network. However, the engagement of these interactive networks, and regions within it (e.g., hippocampus or amygdala) depend on the type and amount of information that needs to be processed at a particular time.

### Mental Simulation Network

Counterfactual thought engages a neural network that supports core processes for mentally undoing the present state of affairs and imagining alternative realities “if only” different decisions were made or actions taken (e.g., Nieuwland, 2012; Urrutia et al., 2012; De Brigard et al., 2013; Kulakova et al., 2013; Van Hoeck et al., 2013, 2014). This mental simulation network, also called the default mode network, engages regions in the medial frontal and temporal lobes, the posterior cingulate cortex, precuneus, and the lateral parietal and temporal lobes. This network is active when individuals are engaged in internally focused tasks, self-projection and scene construction, including autobiographical memory retrieval, envisioning the future, and conceiving the perspectives of others (e.g., Buckner and Carroll, 2007; Hassabis and Maguire, 2009; Spreng et al., 2009; Spreng and Grady, 2010; Summerfield et al., 2010; Schacter et al., 2012, 2015; Spreng and Mar, 2012; Martinelli et al., 2013). Thus, this network is engaged when a situation is observed and interpreted, mentally altered and re-evaluated (i.e., all stages of counterfactual thought). Medial temporal lobe regions, such as the hippocampus, provide information from prior experiences in the form of memories and associations that are the core building blocks of mental simulation. Medial PFC regions facilitate the flexible use of this information during the construction of self-relevant mental simulations. These two subnetworks converge on important nodes of integration including the posterior cingulate cortex. Here we review emerging neuroscience evidence linking counterfactual thought to this network’s function.

Van Hoeck et al. (2010, 2013) directly compared past, future and counterfactual simulations of episodic events (i.e., specific personal experienced events) in this fMRI study. Healthy college students performed three tasks: (1) Recall a negative past event (e.g., “Imagine/recall the car accident from your holiday in France”); (2) Imagine how a similar future event turns out positive instead (e.g., “Imagine a future car trip that goes well”); and (3) Imagine how the past event might have turned out better (a positive upward counterfactual) given a specific additive counterfactual action (e.g., “Imagine what would have happened if you had paid more attention to the road during that car trip in France”). Extensive neural overlap between these tasks was expected and observed. Episodic past, future and counterfactual simulation commonly activated the mental simulation network. But compared to past episodic thinking in particular, counterfactual simulation of episodic events engaged the mental simulation network to a larger extend (see Van Hoeck et al., 2013 for more information). Similar results were also documented by De Brigard et al. (2013), who likewise compared episodic past and counterfactual simulation. This stronger engagement might be understood as counterfactual thinking being a more complex or strenuous form of mental simulation, whereby both factual as counterfactual elements need to be represented and inferred.

A follow-up study conducted by Van Hoeck et al. (2014) demonstrated that counterfactual reasoning about hypothetical social scenarios engage this mental simulation network as well. Participants read a short scenario involving two agents, one of whom moved an object (e.g., moving a wallet from place to place) or changed the content or characteristics of an object (e.g., changing the contents of the wallet). Participants made counterfactual inferences (e.g., what if the object had not been moved?), false belief inferences (e.g., what would the absent agent expect?), and conditional inferences (e.g., what used to be in the wallet?). All three types of inference engaged the mental simulation network, confirming the general role of this network in the construction of mental representations.

During episodic counterfactual simulation the engagement of ventral medial prefrontal cortex (vmPFC) probably supports the construction of the “nearest possible world” by inferring relevant information from prior experience and background beliefs. vmPFC can also integrate the information from the constructed counterfactual event into existing representations, updating prior expectations and action-value associations, which serve to guide future behavior (see vmPFC/mOFC discussion in “Emotion and Value Processing Network” Section). This integration of counterfactual simulations with memory processes can also explain how counterfactual inference can contribute to false memories (e.g., De Brigard, 2013; Gerlach et al., 2013). Indeed, vmPFC involvement during counterfactual simulation may depend on the subject’s engagement in affective, self-reflective inference making (see also D’Argembeau et al., 2013).

We suggest that the hippocampus is more likely to be engaged during counterfactual thinking in situations requiring extensive *arbitrary relational binding* (Nieuwland, 2012; Urrutia et al., 2012; De Brigard et al., 2013; Kulakova et al., 2013; Van Hoeck et al., 2013, 2014). For example, when objects are randomly arranged on a table, the hippocampus will be more critically involved in remembering which object went in which location (location is not an inherent charateristic of an object, since objects can be moved and any could be bound to any location; therefore this arbitrary relationship needs to be encoded), than it would be involved in reconstructing the general gestalt of the figure formed by the objects (this could be mapped to semantic knowledge for “squares” or “triangles” c.f. Watson et al., 2013). Previous work indeed indicates that the hippocampal memory network (e.g., parahippocampal gyrus, fusiform gyrus, perirhinal cortex, vmPFC) is more strongly activated when elements that were previously not related are bound together, for instance, during novel scenario simulation or the generation of new concepts (Hassabis et al., 2007; Addis et al., 2009; Hassabis and Maguire, 2009; Summerfield et al., 2009; Maguire and Hassabis, 2011; Zeithamova et al., 2012; Maguire and Mullally, 2013; Van Mulukom et al., 2013; Watson et al., 2013). During the construction of counterfactual scenarios the hippocampus will be enganged when information about the relations between elements of the scenario cannot be easily inferred from prior knowledge (Henke, 2010). The more changes we make to prior history (e.g., the remembered or experienced event) while creating the counterfacutal representation the more the hippocampus is involved. The hippocampus’ role in scene-construction and relational binding also explains why constructing a future episode, and not always a counterfactual episode (which adheres to the “nearest possible world” constraint), engaged the hippocampal area more strongly than constructing a past episode (Addis and Schacter, 2012; Van Hoeck et al., 2013).

That counterfactual reasoning is strongly constraint by prior knowledge explains the increased engagement of the lateral temporal lobe (Nieuwland, 2012; Urrutia et al., 2012; Van Hoeck et al., 2013, 2014). This is often interpreted as an engagement of semantic processing, using general knowledge to construct the counterfactual scenario rather than information tied to a particular event or arbitrary configuration of elements (Addis et al., 2009; Viard et al., 2011; Irish et al., 2012; Schacter et al., 2012; De Brigard et al., 2013). Counterfactual reasoning that involves many layers of contextual abstraction (Gilead et al., 2012) may engage dorsal mPFC (dmPFC; Scott et al., 2003; Binder et al., 2009; Urrutia et al., 2012; Baetens et al., 2013; Van Hoeck et al., 2013, 2014). Goal-directed behavior, a common component of counterfactual thought, have been shown to engage inferior partietal lobe (IPL) and premotor cortex (Glover, 2004; Gazzola and Keysers, 2009; Van Overwalle and Baetens, 2009; Moody and Gennari, 2010; Stosic et al., 2014). It is worth emphasizing that because the content of counterfactual scenarios varies, so too will the engagement of different brain networks. For example, when there is a strong social component present in the situation we can expect the temporo-parietal junction to be more engaged. This in contrast to when the social component is absent and instead there is a stronger focus on a contextual and goal-directed action component (IPL). Depending upon the level of detail required, the number of relations between elements to configure, and the connection to specific or general events, different counterfactual paradigms will elicit slightly different configurations of the mental simulation network. The contribution of mental simulations to counterfactual reasoning then is in the construction and updating of an accurate representation of the scenario in question. Manipulation, reasoning, and decision making that follow from that representation tend to engage other cognitive control regions, which we turn to now.

### Cognitive Control Networks

Although it has never been the direct focus of a neuroscience study on counterfactual reasoning, we can assume that counterfactual thinking engages cognitive control and reasoning processes, and the outcomes of these processes contribute to overt and covert behavior regulation. We discuss two main control networks, the fronto-parietal network and the cingulo-opercular network, that have been engaged during counterfactual reasoning. We suggest that these networks: (1) enable switching between immediate experiences and counterfactual scenarios; (2) perform the mental transforms required for counterfactual inference and reasoning; and (3) monitor and update representations of the relative values of actions to steer subsequent behavior.

#### Fronto-Parietal Control Network

The fronto-parietal control network consists of the lateral PFC (lPFC), middle cingulate cortex, IPL and precuneus. This network enables the integration of multiple sources of information and supports the regulation and control of thought and behavior (e.g., Miller and Cohen, 2001; Seeley et al., 2007; Dosenbach et al., 2008; Vincent et al., 2008; Nelson et al., 2010; Spreng and Grady, 2010; Shackman et al., 2011; Barbey et al., 2012, 2013; Cabeza et al., 2012; Chein and Schneider, 2012; Schacter et al., 2012; Spreng, 2012; Whitman et al., 2013). The IPL and dorsal lateral PFC (dlPFC) play an important role in simulating and processing counterfactual information (e.g., Coricelli et al., 2005, 2007; Chua et al., 2009; Fujiwara et al., 2009; Van Hoeck et al., 2010, 2014; Nieuwland, 2012; Xue et al., 2012; De Brigard et al., 2013), but this representational role seems to be in service of executive, goal-directed functions. For example, the dlPFC (and posterior parietal cortex) was more strongly activated when processing information related to counterfactual *outcomes*, especially in situations with mixed appraisals (e.g., although you won, you could have won more; c.f. Henderson and Norris, 2013). In addition, the left dlPFC plays a central role in the manipulation of cognitive representations in the service of goal-directed behavior (Barbey et al., 2009, 2013; Ruh et al., 2012).

Knight and Grabowecky (1995; p. 1367) provide evidence from a patient with dlPFC damage who demonstrated a “complete absence of counterfactual expression”. Likewise, Gomez Beldarrain et al. (2005) observed significantly fewer spontaneous counterfactual thoughts in a group of dlPFC and orbital frontal cortex (OFC) lesion patients. However, these patients were able to construct hypothetical scenarios in response to specific cues, emphasizing the distinction between the mental simulation network and the more outcome-oriented, comparative PFC network.

#### Cingulo-Opercular Control Network

The cingulo-opercular network includes the dorsal anterior cingulate cortex (dACC), the posterior medial frontal cortex (pmFC), anterior insula (aI)/frontal operculum (posterior lateral OFC), and the anterior PFC (aPFC). This network is consistently linked to maintaining task goals and monitoring actions, as well as contributing to slow behavioral adjustments over time (e.g., Seeley et al., 2007; Dosenbach et al., 2008; Power et al., 2011; Chein and Schneider, 2012). For example, Boorman et al. (2011) provide evidence that activity within this network (pmFC and aPFC) positively correlates with the reward-probability of a counterfactual choice option. Lateral aPFC activation also correlated positively with the reward-probability of the most favorable counterfactual option, and negatively with worst counterfactual option, suggesting that the lateral aPFC encodes and updates information associated with the best alternative and provides a signal for switching to an advantageous alternative (Boorman et al., 2009, 2011; Rushworth et al., 2011). These results fit well with previous findings of pmPFC involvement in adaptive behavior during tasks that entail performance monitoring, decision related uncertainty, and detection of response conflict and unfavorable outcomes (Ridderinkhof et al., 2004; Danielmeier et al., 2011). Similarly, in an episodic counterfactual simulation task, lOFC and aI activity is associated with increasing evidence of the counterfactual simulation’s probability (De Brigard et al., 2013). This finding helps to explain why lOFC activation increased in a stimuli-identification task when subjects were aware that their upcoming performance would not be rewarded (there are better opportunities/trials out there) and* vice versa* (Ursu and Carter, 2005). Together these results suggest that the cingulo-opercular network critically contributes to behavioral regulation and feelings of regret by updating and maintaining counterfactual action-outcome information and signaling when it becomes profitable to change behavior (Liu et al., 2007; Nicolle et al., 2011b; Hampshire et al., 2012; Rudebeck et al., 2013).

### Emotion and Value Processing Network

We have reviewed evidence to elucidate the neural mechanisms that support the construction of counterfactual scenarios and for evaluating and switching between these nearby possible realties to guide behavior toward rewarding alternatives. We now turn to the question of where these evaluative and rewarding signals originate: the networks underlying emotional and value processing traditionally associated with affective learning, valuation, reward processing, and autonomic/endocrine control (for an extensive overview see Roy et al., 2012). These networks include the vmPFC (including the medial orbitofrontal cortex, mOFC), amygdala, basal ganglia, lOFC and lPFC. We selectively review neuroscience studies that provide insight into how this network supports each of the central affect-related components of the counterfactual reasoning process (see especially the activation and adaptation-stage).

The amygdaloid complex is most frequently associated with emotional arousal and evaluative judgments (Berntson et al., 2011), especially in integrating sensory and associative information to process negative stimuli (e.g., LeDoux, 2000; Dolan, 2002; Ochsner and Gross, 2005; Phelps, 2006; Lewis et al., 2007; Kim et al., 2011; Bzdok et al., 2013; Denny et al., 2013). In addition, while the amygdala is important for reflexive or unconscious emotional processing, it is also involved in judgments of self awareness and personal responsibility (Sander et al., 2003; Li et al., 2011; Nicolle et al., 2011b; Zalla and Sperduti, 2013). For example, Nicolle et al. (2011b) demonstrated that the amygdala responded to receiving the worse outcome from two gamble-options (counterfactual outcome was better) when these were associated with high, vs. low, personal responsibility.

Encoding the *relative value* of the obtained outcome of an event is supported by components of the basal ganglia and aI/plOFC. In a sequential decision—making study that asked subjects to invest money in a market based on its history (D’Ardenne et al., 2013), Blood Oxygen Level Dependent (BOLD) activity in two dopaminergic basal ganglia structures (the substantia nigra and the ventral tegmental area) was positively correlated with the relative values of the obtained outcome (the difference with prior expectations and with the counterfactual outcome). The BOLD response in these regions also tracked the influence of these relative values on subsequent behavior, indicating their contribution to experiential and counterfactual learning.

Further upstream in the dopaminergic pathway, striatum activity has been associated with *approach motivation*: encoding the degree of “wanting” of “liking” particular outcomes, displaying activation when relative outcome values favor an upcoming decision (i.e., no change of decision—making behavior is needed; current plan of action is favorable) and a stronger deactivation with increasing evidence against current action (i.e., change of decision making is needed; alternative/counterfactual actions need to be considered; Büchel et al., 2011; Nicolle et al., 2011a; Palminteri et al., 2012; D’Ardenne et al., 2013; Henderson and Norris, 2013; Tobia et al., 2014). aI/plOFC activity, on the other hand, is associated with sub-optimal evaluations (relative loss or threatening), contributing to feelings of regret and promoting corrective behavior (O’Doherty et al., 2003; Chua et al., 2009; Henderson and Norris, 2013). The aI/plOFC has also been implicated in encoding unanticipated avoidance of negative outcome (relief) and may thus in general contribute to assignment of *evaluative value* (Chandrasekhar et al., 2008; Berntson et al., 2011; Palminteri et al., 2012).

Patients with lesions in frontal regions demonstrate this interplay of action-value associations. Levens et al. (2014) examined plOFC and vmPFC/mOFC lesion patients’ ability to generate reward expectations prior to making a decision and to assign a value to each outcome once the decision was made. Patients selected between two alternative “wheels of fortune” gambles. Each gamble had two possible outcomes (win or lose) with different reward-probabilities. This task doubly dissociated the behavior of patients with damage in the posterior lOFC from patients with vmPFC/mOFC damage. Patients with damage confined to plOFC successfully generated reward expectations and used them to calculate the expected value of each choice but were impaired in processing post-decision feedback in favor of the counterfactual choice option. By contrast, patients with vmPFC/mOFC damage selected financially worse gambles, demonstrating difficulty in computing expected value but their post-decision affect reflected both the true and counterfactual outcomes.

These findings illustrate the distinction between assigning/learning evaluative value (undergirded by aI/plOFC) and the computation of expected values (vmPFC/mOFC). The literature broadly supports vmPFC/mOFC involvement in inferring the affective meaning of a situation by connecting conceptual information (based on personal prior experiences, beliefs, traits, and goals) with its subjective value (e.g., Coricelli et al., 2007; Van Overwalle, 2011; D’Argembeau and Salmon, 2012; Roy et al., 2012; D’Argembeau et al., 2013; Moore et al., 2014; Zaki et al., 2014). Through these value associations, vmPFC/mOFC can represent specific *expectations* about the outcome of an event and *update* these representations in light of new information (e.g., expectancy violations of counterfactual outcome values; O’Doherty et al., 2003; Alexander and Brown, 2011; Tobia et al., 2014). These findings indicate that vmPFC/mOFC abnormalities result in impairments in expectation-based regulation of emotions and behavior (Mellers et al., 1997; Sutton and Barto, 1998; Levens et al., 2014). This is generally consistent with the role of vmPFC/mOFC in the top-down modulation of emotional responses by ascribing affective meaning to the sensory information processed in the amygdaloid complex (Coricelli et al., 2007; Canessa et al., 2009; Kim et al., 2011; Zalla and Sperduti, 2013).

Taken together, these networks provide a basis for the diverse emotional and evaluative processing required during counterfactual thought.

## Clinical Considerations

While these cognitive and neuropsychological studies have begun to treat counterfactual thought as an important part of human reasoning, little is known about how counterfactual thought is altered by psychiatric illness and neurological disease. We now turn to a review of the literature on counterfactual impairments and brain networks in clinical populations, including individuals with psychopathy, depression, schizophrenia and Parkinson’s disease, and propose, in each case, that patients demonstrate systematic impairments in the cognitive and neural mechanisms for counterfactual inference.

### Psychopathy

Psychopathic traits can be parsed into at least two interrelated components, the affective-interpersonal features (Factor 1; e.g., callousness, lack of empathy or emotional depth, and lack of genuine remorse) and the impulsive-antisocial traits (Factor 2; e.g., aggression). The former Factor one traits distinguish psychopathy from other antisocial syndromes and make people with such traits particularly pernicious (e.g., prone to repeated violence despite sanctions).

Adults with psychopathic traits display normal executive functions but decreased arousal for aversive stimuli and aberrant moral behavior (Koenigs et al., 2012; Young et al., 2012), possibly exacerbated by abnormalities in selective attention: ignoring elements outside their central focus of attention (early bottleneck; Baskin-Sommers and Newman, 2013). In addition, Blair (1995) suggests that because individuals with psychopathic traits lack the emotional responses that lead ordinary people to imbue moral rules with genuine, authority-independent moral legitimacy, they fail to distinguish between moral and conventional rules and see all rules as mere rules. This indicates that, especially during goal-pursuit (focused attention), immoral behavior will not produce regular emotional arousal and/or will be evaluated differently. These affective and moral value related deficits have been connected with abnormal shape, size, activity and connectivity of regions of the emotion and value processing network and of the cognitive control networks (Blair, 2007; Glenn et al., 2009, 2012; Koenigs et al., 2011; Motzkin et al., 2011; Koenigs, 2012; Marazziti et al., 2013).

Within a framework of counterfactual thought, we argue that adults with psychopathic traits demonstrate deficits primarily attributable to abnormalities within the emotion and value processing network chiefly related to amygdala and vmPFC. These individuals are likely to exhibit decreased arousal for negative stimuli and abnormal selective attention (reducing the saliency of a rule violation), which will avert the activation of counterfactual inference. Individuals with psychopathic traits may assign a different affective meaning to immoral behavior, impacting the content of the counterfactuals they construe (e.g., focusing on the victim). As a result of a reduced spontaneous elicitation of counterfactual thought and different counterfactual content, they may report less remorse and self-blame and correct their immoral behavior less frequently. We predict that these deficits will exist in the presence of largely intact mental simulation and executive processes, except where those processes interact with the impaired moral or emotional processing.

### Depression

Depression is a mood disorder characterized by feelings of sadness, apathy and ruminative thoughts (Nolen-Hoeksema, 2000). Rumination shares many features with counterfactual thought. Rumination can be described as a stable thought process that is repetitive, general, self-focused and oriented toward negative emotions whereby the trigger and content likely involve the discrepancy between the desired and actual state of affairs (Nolen-Hoeksema, 2000; Nolen-Hoeksema et al., 2008; Watkins, 2008; Smith and Alloy, 2009). However, it is still unclear how rumination and counterfactual reasoning are related to each other (many rumination measures include “regret”—a counterfactual emotion- as an item). There is some evidence speaking to the mood related changes occurring during counterfactual thought in individuals with depressive symptoms. A study conducted by Quelhas et al. (2008; Experiment 2) demonstrated that, immediately after receiving a negative grade on an academic test, college students with moderate symptoms of depression (measured by Beck Depression Inventory [BDI];* M*_BDI–II_ = 18.95) generated a smaller number of *spontaneous* counterfactual thoughts relative to controls (*M*_BDI–II_ = 3.08). This difference was eliminated by an *explicit cue* to generate upward counterfactuals regarding a recent negative academic experience (Markman and Miller, 2006). In addition, upward counterfactuals generated by individuals with symptoms of depression and controls, had similar levels of focus on controlling the situation by changing one’s own behavior. What differed was the feasibility of counterfactuals generated by the depression group: their alternative scenarios included less plausible actions and/or actions less likely to change the outcome. Depression *severity* also influenced number of counterfactuals focused on chronic or enduring aspects of the self (which are unlikely to change or be under immediate control). In addition, consistent with the earlier statement that processing upward counterfactuals with low feasibility only contributes to psychological distress but does not result in beneficial reappraisal effects, the post-counterfactual mood of depression groups was more negative than controls. Moreover, subjects with mild-to-moderate symptoms (BDI-II cutoff: 10–23) did not perceive an increase in their ability to exert control over the environment (see also Quelhas et al., 2008) and in the severe group (BDI-II cutoff: 24+) the perception of control decreased and re-evaluation of the event produced a more negative assessment than before the counterfactual thinking.

The altered spontaneous counterfactual inference pattern in depression might be related to other irregularities associated with depression, including deficits in removing negative stimuli from short-term memory (Joormann and Gotlib, 2010) and decreased attentional control when confronted with self-relevant negative information (De Raedt et al., 2010; Koster et al., 2011; Beckwé et al., 2013), which might prevent them from disengaging from their immediate emotional experience and ruminative thought and reallocating resources and attention to the (next) counterfactual inference process of how the event could have unfolded differently (Dolcos et al., 2008; Iordan et al., 2013). In addition, their rumination tendencies and overgeneral autobiographical memory may also contribute to generating counterfactuals that focus stronger on enduring traits than on specific behavior (Williams, 2006; Williams et al., 2007; Watkins, 2008; Sumner, 2012).

Depression may also impact processing of counterfactual information when the experimenter provides this information, rather than it being a product of an individual’s own counterfactual construction. Subjects with mild symptoms of depression (*M*_BDI_ = 14.66) reported more regret about a *hypothetical* hiring decision than controls when they were informed about another, better applicant (Monroe et al., 2005). This increase in regret was not driven by the absolute difference in applicant quality or by the individual’s initial confidence in the hiring decision. Howlett and Paulus (2013) attributed this increased experience of regret in subjects with mild-to-moderate symptoms to: (1) an increased vigilance for potential threats to self-esteem, indicating that negative stimuli are associated with a high self-relevancy and thus increase their affective meaning; (2) a stronger decrease of the relative value of the obtained outcome; and (3) increased damage to self-esteem when considering a better counterfactual alternative.

However, major depression disorder displays a different emotional profile, wherein sensitivity to relative losses (e.g., one could have won more) is muted and this response is related to self-reported apathy-scores (Chase et al., 2010). This might reflect changes in approach motivation and goal-processing (Shankman et al., 2007; Eddington et al., 2009), and suggests that this disengagement might be related to coping mechanisms developed to protect their unstable self-esteem (Roese et al., 2009; Howlett and Paulus, 2013). This pattern of results might also be linked to the finding that individuals’ with severe symptoms of depression, when cued to generate counterfactual scenarios, tend to construe less feasible, non-beneficial upward counterfactuals (focused on chronic or enduring aspects of the self instead of actions that would likely change the outcome), and experience afterwards a more negative mood than controls (Markman and Miller, 2006). In this case, disengaging from counterfactual reasoning may prevent increased psychological distress (Wrosch and Miller, 2009; Brassen et al., 2012; Wrosch et al., 2013), but may also prevent improvement from status-quo (see Tykocinski and Steinberg, 2005).

The literature on the neurobiology of depression is complex, with differences related to severity, time of onset, and chronicity. Changes are most consistently observed in emotion and value processing regions, such as amygdala, vmPFC, striatum and OFC (Johnstone et al., 2007; Stein et al., 2007; Eddington et al., 2009; Johnson et al., 2009; Townsend et al., 2010; Murray et al., 2011; Price and Drevets, 2012; Howlett and Paulus, 2013; Cole et al., 2014). Depression has also been associated with increased connectivity between and alterations within the aforementioned mental simulation network (e.g., Zhang et al., 2011). For example, severity of depression correlates positively with activity in ventromedial polar cortex and amygdala, possibly representing an increased vigilance for potential threats to self-esteem (Drevets, 2007; Murray et al., 2011; Price and Drevets, 2012). On the other hand, activity within the posterior OFC, a region we identified as playing a key role in processing counterfactual action-outcome information, decreases with severity. This finding is largely consistent with data from posterior OFC lesions patients indicating that damage to this region influences expectations about their post-decision affect but not for the counterfactual outcome (Levens et al., 2014).

In summary, depression symptoms seem to interfere with all three aspects of counterfactual thought: mental simulation, executive control and valuation. This interference is complex, and interacts with severity, chronicity, and onset of time of depression. Disruption is especially apparent for negative and self-relevant judgments, such as disengaging from self-relevant negative experiences. However, looking at depression symptoms through a lens of counterfactual thinking helps to identify disruptions that would not be immediately apparent from an evaluation of depression as a mood disorder alone. For example, subjects’ generation of less feasible, likely, or detached counterfactuals is highly characteristic of depression but not closely linked to mood. Counterfactual thought can therefore provide a useful technique for understanding depressive pathology and symptoms.

### Schizophrenia and Parkinson’s Disease

Schizophrenia and Parkinson disease patients both exhibit primary deficits in executive functions and social cognition, stemming from disruptions within dopaminergic pathways in the brain. We discuss similarities in the impairments of counterfactual thought exhibited by these clinical groups.

Schizophrenia, a psychopathology with significant neurological dysfunction and symptoms including auditory hallucinations, paranoia, and other delusional thoughts, is also known to impair counterfactual thinking. Hooker et al. (2000) had 14 residential Schizophrenia patients (11 males and 3 females taking antipsychotic medication; with no history of substance abuse, head injury or co-morbid Axis one disorder) recall a negative personal event from the past year. They were explicitly asked if, as they recalled the past, they had any thoughts about how this event could have turned out differently. In comparison to healthy controls, schizophrenia patients mentioned fewer different counterfactual thoughts (see also Caño et al., 2011). Their performance could not be explained by a generalized cognitive deficit: in comparison to the control subjects they did not differ on Wechsler Adult Intelligence Scale (WAIS-R) measure of Vocabulary and Digit Span, or on the FAS Verbal Fluency test (verbal production). Key components for counterfactual simulations are broadly impaired by Schizophrenia. Research on episodic simulation indicate that these patients are impaired in the coherent recall of episodic events, inferring self-relevant meaning, and (re)construction of episodic future scenarios (D’Argembeau et al., 2008; Raffard et al., 2010a,b; Bennouna-Greene et al., 2012).

In addition, deficits in cognitive control may aggravate these problems (Cole et al., 2014). Savla et al. (2012) examined the performance of patients on measures of cognitive flexibility and abstraction (Silver and Bilker, 2013). Decreased cognitive flexibility was related to increase of positive symptoms associated with schizophrenia. This factor also includes failures of inhibition and control of attention (Clark et al., 2010; Goldberg et al., 2011; Waters et al., 2012). Furthermore, abstraction correlated negatively with the duration of illness and positively with everyday functioning. These results suggest broad impairments in counterfactual thought involving both failures to simulate and regulate counterfactual scenarios.

Consistent with this pattern of results, Schizophrenia patients display impairments the Counterfactual Inference Test (CIT; Hooker et al., 2000; Larquet et al., 2010). The CIT assesses social cognition across four vignettes, each of which describes a negative event in which two protagonists differ in their proximity to a better outcome or the normality of the action preceding the negative outcome (routine vs. uncommon action). To illustrate, consider the following example. “Janet is attacked by a mugger only 10 feet from her house. Susan is attacked only a mile from her house. Who is more upset by the mugging? (a) Janet (b) Susan (c) Same/Cant’ tell” (Hooker et al., 2000, p. 330). Subjects were asked to judge whom of the two protagonists felt worse after experiencing this event, regrets their action the most, or thinks most (counterfactually) about the event. Schizophrenia patients were near chance in selecting appropriate CIT answers. Their performance could not be explained by a generalized cognitive deficit (WAIS-R: Vocabulary and Digit Span; FAS Verbal Fluency test). However, performance on CIT did mediate the group difference in social competence (measured by the Zigler scale). In addition, patients showed decreased activity in brain networks associated with reasoning about the cognitive states of others (i.e., Theory of Mind; temporo-parietal junction, mPFC and lower portion of the precuneus; Amodio and Frith, 2006; Van Overwalle and Baetens, 2009; Mar, 2011; Schilbach et al., 2012) during completion of false belief tasks (which require inferring others’ representations of reality, which does not correspond with their own perception reality; Lee et al., 2011). The observed pattern of findings suggest that Theory of Mind is not in general impaired in Schizophrenia, but that it instead reflects deficits in reasoning when complex non-factual elements are needed to understand the social environment (Kern et al., 2009; Sparks et al., 2010). Both counterfactual and false belief inference entail simulation of an alternative state of affairs different from the perceived reality. Van Hoeck et al. (2014) further confirmed that false belief and counterfactual inference about social hypothetical scenarios activate the mental simulation and frontoparietal networks (see also Canessa et al., 2009). These authors further suggest that additive counterfactual inference may reflect a more complex process that places additional demands on cognitive control (increased activation in dlPFC and pmFC).

An abundance of neuroscience studies confirm abnormalities within the simulation and cognitive control networks, especially those regions supporting contextual coherence, relational processing and flexible cognitive control. For example, structural, functional, and neurochemical hippocampus abnormalities have been associated with Schizophrenia, therefore contributing to observed problems in scene-construction and relational processing during episodic simulation (Ongür et al., 2006; Boyer et al., 2007; Williams et al., 2010; Adriano et al., 2012; Herold et al., 2013). dmPFC, a region involved in contextual associations/abstractions and contributing to the coherency of the mental representation, shows altered interactions with vmPFC and posterior midline structures in Schizophrenia (Raffard et al., 2009; Shad et al., 2011; Alonso-Solís et al., 2012), and also decreased anticorrelation with the dlPFC, a frontoparietal control component (Whitfield-Gabrieli et al., 2009).

Similar disruptions are found in Parkinson patients. Parkinson is a progressive neurodegenerative disorder associated with substantial decrease in dopamine levels in the basal ganglia, frontal lobes and hippocampus. As in the case of Schizophrenia, Parkinson patients demonstrate Theory of Mind dysfunction (especially with respect to the cognitive component; Yu and Wu, 2013), impaired cognitive flexibility, selective attention and concept formation (Kudlicka et al., 2011). Parkinson patients, in comparison to age-matched controls, also generate a smaller number of upward episodic counterfactual thoughts and demonstrate decreased performance on the CIT (at chance level; McNamara et al., 2003). Their counterfactual performance could not be explained by a general cognition deficit, but did correlate with social function and cognitive flexibility (as assessed by Stroop color inference task: inhibition, resistance to cognitive interference; Tower of London task: ill-structured problem space requiring planning and working memory).

Thus, evidence from psychology and neuroscience demonstrate impairments in the mental simulation and executive function components of counterfactual thought in Schizophrenia and Parkinson disease. Both patient groups demonstrate impairments in cognitive flexibility, contextual abstraction and relational binding. These deficits are especially apparent within complex, ill-structured environments, and in the context of goal-directed behavior and adaptive decision making. Since all of these functions are required in counterfactual thought, tests of counterfactual thinking may, once again, serve as a valuable diagnostic tool.

## Conclusion

Counterfactual reasoning entails three processing stages: Activation, Inference and Adaptation. Counterfactual scenarios are automatically constructed in response to everyday events and are linked to violations of prior beliefs and motivations, adhering to a “nearest possible world” constraint. These mental simulations influence our emotional responses and future behavior, by integrating information about the counterfactual state of affairs into the representation of the current situation, prior beliefs, and expectations thereby allowing one to learn through counterfactual inference. This complex psychological phenomena of counterfactual reasoning is made possible by the coordinated interaction between three networks: (1) mental simulation in MTL/PFC; (2) cognitive control in the fronto-parietal and cingulo-opercular network; and (3) motivation and valuation in limbic regions and vmPFC.

Because counterfactual reasoning depends upon so many inter-related brain systems and mental processes, it provides a broad window onto global brain function. Disruptions to counterfactual reasoning are a productive scientific and diagnostic tool. By identifying the specific sub-components of counterfactual reasoning that are affected clinicians can better understand the day-to-day challenges faced by different patient populations. In our everyday lives, counterfactual reasoning is a ubiquitous source of new insights into nearby possible worlds, and different circumstances, scientific understanding of this process is a critical component of insight into the complex alternate worlds that make up our rich, inner lives.

## Conflict of Interest Statement

The authors declare that the research was conducted in the absence of any commercial or financial relationships that could be construed as a potential conflict of interest.
